# Supplementary material for: Wide reference databases for typing Trypanosoma cruzi based on amplicon sequencing of the minicircle hypervariable region
Source: PLoS Negl Trop Dis. 2023 Nov 13;17(11):e0011764. doi: 10.1371/journal.pntd.0011764 (PMC10681310; doi:10.1371/journal.pntd.0011764)
Supplement: S1 Table — (PDF) [file pntd.0011764.s001.pdf]

Supplementary Table 1. Initial reads and reads retained after different steps in the workflow.

	Strain	DTU	Initial reads	Quality-filtered and merged reads	Retained reads after quality filter
1	LL0553R2cl3	TcI	69414	67697	63230
2	PalDa20cl3	TcI	2728193	2545361	2356494
3	PalDa30V2cl2	TcI	52980	51475	47996
4	PalDa4	TcI	53430	52077	48791
5	TeDa2cl4	TcI	1113033	1113033	950142
6	TEV55cl1	TcI	2305460	1366928	1202355
7	86/2021	TcI	72526	70068	64859
8	P209cl1	TcI	130704	127081	117591
9	QRA05	TcI	154003	149267	138019
10	SO40	TcI	43872	43226	40886
11	CUICAcl1	TcI	38049	37492	35325
12	CUTIAcl1	TcI	78631	76727	71977
13	SilvioX10/7	TcI	65063	63030	58739
14	SP104cl1	TcI	139933	135729	126454
15	Vincho111	TcI	32097	31539	29809
16	VQUI1	TcI	42751	42025	39516
17	393TA	TcI	79554	77135	70795
18	Colombiana	TcI	110795	108052	101052
19	MR-C	TcI	78904	76762	71008
20	NS	TcI	22364	21892	20526
21	ElSalvador1980	TcI	82733	80450	75250
22	R143	TcI	56055	54067	50210
23	DAVIS	TcI	49015	47583	44251
24	ARMADILLO1973	TcI	128192	124362	115156
25	DM28c	TcI	96366	92879	85841
26	Saimiri4a	TcI	20342	19825	18207
27	TU18cl93	TcII	2307362	2143482	2000770
28	Bug2150	TcII	68968	67073	63076
29	Bug2152	TcII	114905	111563	104117
30	Esmeraldo	TcII	2328934	1338561	1201724
31	MAS1cl1	TcII	53174	52204	48922
32	X-300	TcII	171865	166979	154040
33	CBBcl4	TcII	104255	102168	96012
34	IVVcl4	TcII	27775	26999	25037
35	LL0513R2	TcIII	150122	145902	135643
36	LL051P24RI	TcIII	135155	129491	119503
37	M5631cl5	TcIII	121017	115871	108442
38	M6241cl6	TcIII	31710	30559	28104
39	X109/2	TcIII	2551201	1651245	1492887
40	CANIIIcl1	TcIV	1450910	1364041	1267374

41	92122102R	TcIV	102936	97384	89407
42	93053102Rcl3	TcIV	31254	30191	28068
43	DogTheis	TcIV	98341	94264	86290
44	STC10Rcl3	TcIV	41543	39955	37035
45	STC13Rcl3	TcIV	94592	90454	82819
46	STC16Rcl4	TcIV	23007	22352	20974
47	STC5Rcl2	TcIV	80517	77606	71253
48	LL014R1	TcV	2003702	996499	827756
49	LL0401R0cl1	TcV	74234	71844	67592
50	SC43cl1	TcV	141358	136738	128727
51	MIz02	TcV	106834	104970	99467
52	CHUL23	TcV	157455	153008	142383
53	Bug2145	TcV	266531	255131	238605
54	MNcl2	TcV	2442318	1161607	982028
55	SAXP19	TcV	127350	123419	115832
56	LL015P68R0cl4	TcVI	2192191	2052980	1895845
57	TeP6	TcVI	124731	121809	114433
58	TeV67	TcVI	58500	57248	53820
59	VM09	TcVI	177724	172405	161777
60	CLBrener	TcVI	141358	28229	26409
61	Tulacl92	TcVI	210211	202501	187503
62	P63cl1	TcVI	67912	66294	62161
