# Supplementary material for: Wide reference databases for typing Trypanosoma cruzi based on amplicon sequencing of the minicircle hypervariable region
Source: PLoS Negl Trop Dis. 2023 Nov 13;17(11):e0011764. doi: 10.1371/journal.pntd.0011764 (PMC10681310; doi:10.1371/journal.pntd.0011764)
Supplement: S2 Table — (DOCX) [file pntd.0011764.s002.docx]

Table S2. True and false-positive rates for different reference sets on reads and strains.

|  |  | Pairwise identity threshold (%) | | | | | |
| --- | --- | --- | --- | --- | --- | --- | --- |
|  |  | 85 | 87.5 | 90 | 92.5 | 95 | 97.5 |
| Reads | Total Reads | 18401312 | 18386217 | 18308287 | 18366069 | 18288722 | 16596532 |
|  | True-positive rate | 0.78 | 0.76 | 0.75 | 0.69 | 0.70 | 0.65 |
|  | False-positive rate | 0.0059 | 0.0048 | 0.0034 | 0.0022 | 0.0016 | 0.0003 |
|  | Non-assigned | 0.219 | 0.230 | 0.242 | 0.306 | 0.297 | 0.352 |
| Strains | True-positive rate | 1 | 1 | 1 | 1 | 0.984 | 0.919 |
|  | False-positive rate | 0 | 0 | 0 | 0 | 0.016 | 0.016 |
|  | Non-assigned | 0 | 0 | 0 | 0 | 0 | 0.065 |
