# Supplementary material for: Wide reference databases for typing Trypanosoma cruzi based on amplicon sequencing of the minicircle hypervariable region
Source: PLoS Negl Trop Dis. 2023 Nov 13;17(11):e0011764. doi: 10.1371/journal.pntd.0011764 (PMC10681310; doi:10.1371/journal.pntd.0011764)

## Evaluation of the PCR simulating algorithm

A PCR simulation algorithm was developed in R (https://github.com/ntomasini/cruzityping/) to simulate PCR amplification. The goal was to simulate how PCR generates stochasticity in the abundance of mHVR clusters (cluster size). A simple way to address PCR stochasticity is to calculate Pearson's correlation coefficient (r) in the mHVR cluster abundances for two independent PCRs on the same sample. To address the suitability of the algorithm for describing the stochasticity of PCR amplification, we compared the variation in r between simulated pairs and two different replications of real PCR (and sequencing) for the LL015P68R0cl4 strain previously published (21). Briefly, the average mHVR cluster size between both PCRs was used to simulate 200 replication pairs with different PCR efficiency. Clusters with an average of less than 30 reads were discarded to reduce sequencing noise, and cluster size was normalized to 30 reads = 1 DNA molecule. The x-axis represents the mHVR clusters that were ordered based on the average cluster size (green line) between the real PCRs with a logarithm scale. The r-coefficient was then calculated along a sliding window of fifty clusters. The figures show a comparison between the r in real PCR repetitions (blue line) and the range between the 2.5% and 97.5% percentiles (shaded yellow) for the simulations for different efficiency values. As expected, r increased with mHVR cluster size (i.e., lower correlation, which indicates higher stochasticity, was observed for mHVR clusters with lower size). Models with an efficiency of 0.8 to 0.9 fitted very well the data. Instead, efficiencies lower than 0.8 simulates higher stochasticity than the observed.

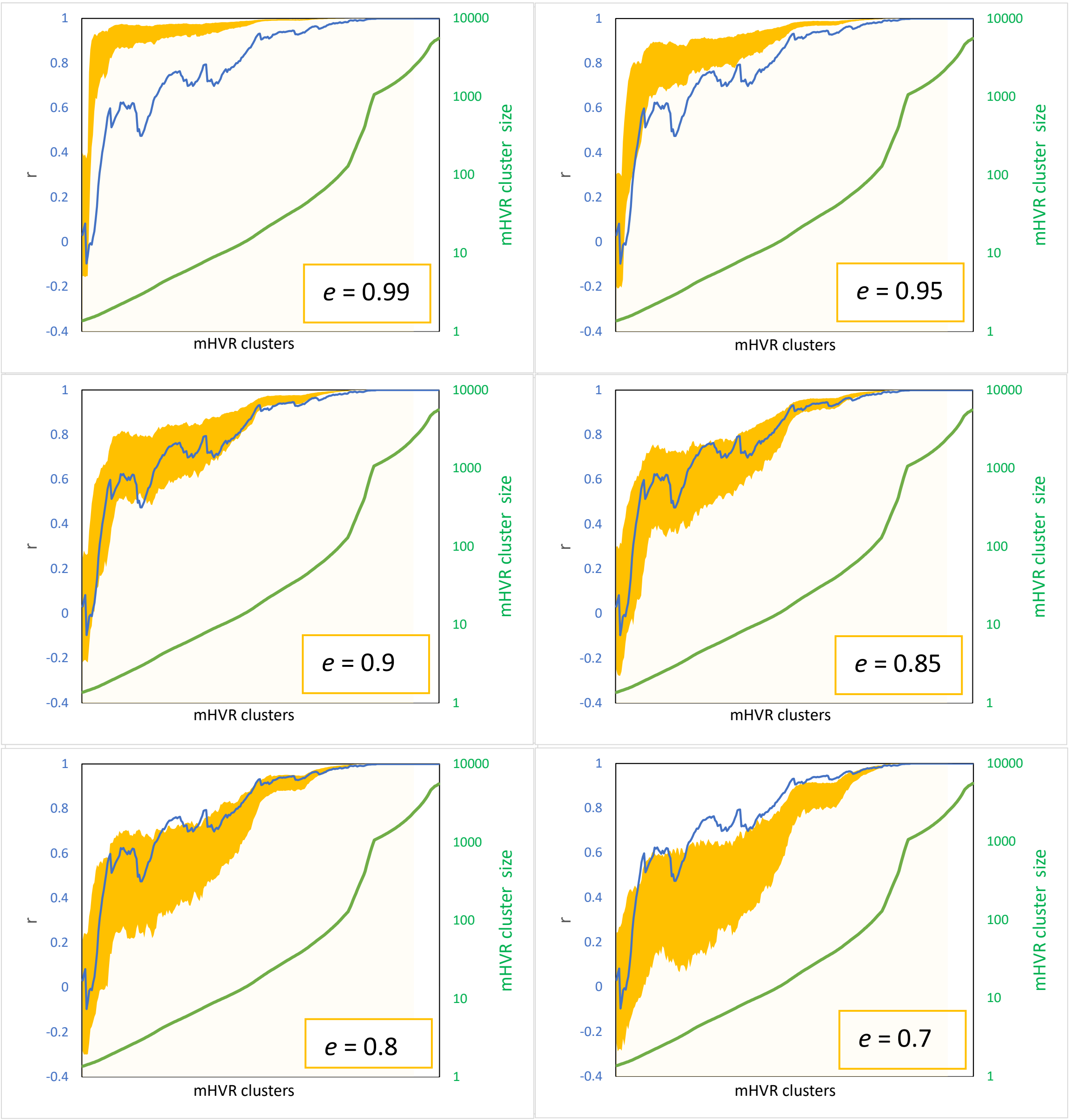

Supplement: S1 File — (PDF) [file pntd.0011764.s004.pdf]
