# Supplementary material for: Wide reference databases for typing Trypanosoma cruzi based on amplicon sequencing of the minicircle hypervariable region
Source: PLoS Negl Trop Dis. 2023 Nov 13;17(11):e0011764. doi: 10.1371/journal.pntd.0011764 (PMC10681310; doi:10.1371/journal.pntd.0011764)
Supplement: Attachment — Submitted filename: responsetoreviewersPNTD.docx [file pntd.0011764.s007.docx]

Response to editor’s comments

As pointed out by the reviewers, the major discrepancies in the scope of the manuscript need to be addressed, from the generation of a database suggested in the title, but not presented and rather proposed as a follow-up work later in the discussion, to the claim of a new typing method/strategy, but no experimental validation with biological samples is presented. The additional comments from the reviewers should also help improve the study.

We extend our gratitude to the reviewers for their thorough and thoughtful comments regarding our manuscript. The opportunity to clarify our work is highly valued, and we would like to provide the following detailed responses:

1) **Database Generation:** Our methodology involved the analysis of approximately 24 million reads from amplicon sequencing of the minicircle hypervariable regions (mHVRs) across 62 strains of *T. cruzi*. The construction of reference databases for each DTU is a central to our proposition. We recognize that the manuscript may not have made this entirely clear, and we appreciate the opportunity to rectify this oversight. In the revised version, we have ensured that these databases are highlighted more prominently, demonstrating their integral role in our research.
2) **Validation and Refinement of the Typing Strategy:** We acknowledge the concern regarding the experimental validation of our proposed typing strategy using biological samples. In the revised manuscript, we will detail the refinement of our method, including its application to patient blood samples, and a comparison with the Southern blot method. It is important to emphasize that this validation was performed against another technique based on the same target (mHVRs). This was made given the challenges in amplifying other commonly used molecular targets, such as SL-IR, in the analyzed samples. Additionally, the absence of a current gold standard for determining *T. cruzi* genotypes in biological samples complicates the traditional validation. However, our work aims to provide a novel method to address this gap, and we believe has been achieved in our study.

Thank you once again for the constructive feedback. We are confident that these changes will strengthen our contribution to the field. Below, you can find detailed responses to the reviewers’ questions and comments.

Response to reviewers

Key Review Criteria Required for Acceptance?

As you describe the new analyses required for acceptance, please consider the following:

Methods

-Are the objectives of the study clearly articulated with a clear testable hypothesis stated?

-Is the study design appropriate to address the stated objectives?

-Is the population clearly described and appropriate for the hypothesis being tested?

-Is the sample size sufficient to ensure adequate power to address the hypothesis being tested?</br>

-Were correct statistical analysis used to support conclusions?

-Are there concerns about ethical or regulatory requirements being met?

Reviewer #1: The objectives and hypothesis are clearly stated in the manuscript and are both evaluated through an appropriate experimental design and strain population.

While the species Trypanosoma cruzi is highly diverse, the molecular typing methods studied so far have not been sufficient to describe the intra-DTU variability, thus hindering the evaluation of the association of genetic variants with clinical-epidemiological variables.

The methodology is detailed both in the population description. The wet lab methodology and particularly the data analysis is clearly described and elaborated.

We acknowledge your thoughtful and encouraging comments. We greatly appreciate your recognition of the clarity of our objectives, hypotheses, and methodologies.

Reviewer #2: 0. Abstract

Do not use abbreviations in the abstract (mHVR) or if at all necessary please use the complete description first, e.g. "minicircle hypervariable region (mHVR)".

Corrected: “The high diversity and copy number of the minicircle hypervariable region (mHVR) makes it a viable target for typing.”

1. Not serious. But still major. The manuscript title starts with "Wide reference databases..." and then the abstract follows with "We present reference databases of mHVR sequences ..." however there is **apparently** no data or database accompanying the manuscript, nor a file deposited anywhere (data dryad, zenodo, figshare).

However, it seems that the data and code is available only in the github repository mentioned in methods (lines 138-139). It is a little hidden gem in the Ms. There is also a nice python notebook. Maybe the authors like to increase the visibility of these resources? I believe these are the major outputs and would increase the impact of the paper and provide a fast route for readers so they can go from sequencing to typing. It is in the interest of authors to make this as easy to use and apply as possible!

Maybe add a webpage (in github?) with a tutorial on how to use the notebook and these databases? Provide these in the Ms in a more prominent section (DATA AND CODE AVAILABILITY or maybe better REFERENCE DATASETS AND CODE). In any case, you get the idea, currenly the fact that the database is inside the github repo gets lost in the methods, and also the github repo is only mentioned at the end of the "Using the reference datsets".

We appreciate your comment on the accessibility of the reference databases mentioned in our manuscript. We apologize for the oversight and confusion that may have been caused.

In response to your concern, we would like to clarify that the representative sequence sets that constitute the reference database against which new sequences are compared are indeed uploaded to GitHub. To improve accessibility, we created a webpage for easy access to these datasets, and a link to the webpage is provided in the manuscript and the abstract. Furthermore, weensured that the Google Colaboratory (Colab) provided for the analysis was set up to automatically use these databases.

The web page (https://ntomasini.github.io/cruzityping/) has a tutorial and video tutorial to use the Google Colab tool. In addition, the webpage also has links to Google Colab and to reference databases. Finally, a new materials and methods section was added to this new version of the manuscript to highlight the Reference Databases and the website:

## *Reference datasets availability and online typing tool*

*The two reference datasets of mHVRs, generated at 85% and 95% identity thresholds, are accessible at* [*https://ntomasini.github.io/cruzityping*](https://ntomasini.github.io/cruzityping)*. The methodology outlined in the preceding section was automated through a Google Colaboratory notebook, also available at the aforementioned link. This notebook is configured to accept raw data input, execute the described workflow automatically using reference datasets, and generate various graphical representations. An accompanying tutorial was provided to aid users in navigating this too.*

2. PCR simulation

Methods, page 14, lines 175-203

Is there any reference for the algorithm simulating the PCR? What is the value of e in formula (2) (line 188). In the description the value of m(i) is obvious, as well as the value of n(i-1). However the value of e is described as "the duplication probability of each DNA molecule determined according to the PCR efficiency", but why did you choose the values in line 203 for the PCR efficiency (0.7, 0.8, 0.99)? Please clarify. Is there any published paper describing this type of simulation? Why these values? Please provide references.

We acknowledge the reviewer for pointing this out . The algorithm for simulating the PCR was based on the algorithm proposed by Ruijter et al. (40) with some modifications. First, a multinomial distribution was used to simulate the mix of different DNA sequences in the sample, based on the cluster abundance in each strain. In addition, the efficiency is not considered as the proportion of molecules that are replicated in each cycle. Instead, we added additional complexity by considering the number of molecules replicated at each cycle as a variable with a binomial distribution determined by efficiency. Consequently, efficiency is the probability of molecule replication instead of the proportion of molecules that replicate at each cycle.

The following paragraph has been modified in the manuscript for clarification:

In addition, to evaluate the potential suitability of this workflow for typing biological samples, a PCR simulation algorithm was developed in R (https://github.com/ntomasini/cruzityping/blob/main/VirtualPCRcode.R) to simulate the stochasticity of PCR amplification. The algorithm was based on the basic equation of PCR kinetics proposed by Ruijter et al. (40) but considering the efficiency (*e*) as a probability of molecule replication instead of a fixed proportion of replicated molecules.

In the absence of inhibitors in the sample, PCR efficiency is often higher than 90%, as described in *(41*). Consequently, we used e =0.99 (good efficiency) and we selected two other efficiencies (e=0.7 and e=0.8) to consider PCR inhibition. We have modified the methodology section to clarify this matter:

*Different m_0_ values (1, 10, and 100 starting DNA molecules) were evaluated. In addition, PCR efficiency is commonly higher than 90% but can be lower in the presence of inhibitors (41), and different values for e (0.7, 0.8, and 0.99) were evaluated to simulate optimal and sub-optimal conditions, which may introduce more stochasticity in cluster abundances.*

Have this algorithm been validated experimentally by the authors and/or by others?

We have now included a Supplementary File 1 with an experimental validation of the algorithm by comparing the observed and predicted stochasticity of PCR. Briefly, the observed stochasticity was determined using two independent PCRs (and sequencing) of the same strain (LL015P68R0cl4). The stochasticity for different cluster sizes can be addressed using the Pearson correlation coefficient (r) calculated by comparing the abundance of mHVR clusters between different PCR repetitions. The higher the *r,* the lower the stochasticity in PCR. In addition, it is expected that stochasticity (and *r*) varies for different cluster sizes. Consequently, the mHVR clusters were ordered based on cluster size. Subsequently, *r* was calculated along a sliding window of 50 clusters. As expected, r diminished (stochasticity was higher) for low- abundance mHVR clusters. The procedure was repeated for simulated PCR with different efficiencies and compared to the observed variation in *r*. The variation in *r* for different PCR repetitions of the same strain was well explained by simulated PCRs with e = 0.8-0.9. We have modified the following paragraph in the methodology section:

*Different m_0_ values (1, 10, and 100 starting DNA molecules) were evaluated. In addition, the PCR efficiency is commonly higher than 90%, but it can be lower in the presence of inhibitors (41) and different values for e (0.7, 0.8, and 0.99) were evaluated to simulate optimal and sub-optimal conditions which introduce more stochasticity in cluster abundances. The PCR model was compared to empirical data of mHVR cluster abundances from two independent PCR reactions of the same sample (Supplementary File 1).*

3. Results - Threshold similarity percentages

Why were these two values chosen? (85%, 95%). What was the rationale for these? In the manuscript there are only vague explanations.

Methods, (line 109) "Then, sequences were clustered at 85% and 95% pairwise identity..." Why these thresholds?

Results, (lines 228-229) "The reads were clustered according to sequence similarities using different minimum similarity percentages (85% and 95%). [...] number of shared clusters among strains decreased when higher similarity thresholds were used. For the 85% similarity threshold, it was observed that TcI lineage strains, which were geographically closer, shared more mHVR clusters than strains isolated at greater geographical distances. Additionally, at the 95% similarity threshold, most TcI strains shared a few mHVR clusters. In contrast, the TcV and TcVI strains still shared mHVR clusters with other strains of the same DTU. These results suggest that mHVR sequences can be used for typing T. cruzi strains."

Have you considered running the analysis over a range of similarity thresholds (e.g. 70% to 99% in steps of 1%, 2%, 5% don't know what would be a sensible step size but you get the idea), and measure some metric (sensitivity/specificity) to find the best threshold? Maybe the Area Under the ROC curve (AUC)? Maybe the authors already did this? In this case please provide these data (supplementary) as support for these chosen thresholds.

Please kindly refer to the responses provided below about the used thresholds.

Results, page 18, 95% reference set for typing strains from whole-genome sequencing data. (lines 264-276).

Why was this threshold used? I'm trying to make sense on how to move from "Reference sets constructed with a 95% similarity threshold failed to typify one TcIII strain (M5631cl5)." (lines 253-254, previous section) to "To address the suitability of the 95% mHVRs reference set for typing..." (lines 264-265, beginning of next section). Maybe put out loud and clear what is the rationale behind this? I am a little bit confused because although a stricter 95% reference set is used (perhaps aiming to match at the sub-DTU level?), Figure 3 (and Supp Fig 1A) only show mapping (typification) of strain data (from genome sequencing projects) onto DTU-level groups (same as done with the 85% reference set).

We acknowledge the comments on threshold selection. This was based on a previous paper of our group (21), in which we evaluated different thresholds for clustering mHVRs. We have modified this sentence in the first section of Results section:

*The reads were clustered according to sequence similarities using different minimum similarity percentages (85% and 95%) as in a previous work (21).*

In addition, we have included in this new version of the manuscript the evaluation of different thresholds for typing from 85% to 97.5% in 2.5% increments. The 85% had better sensitivity for DTU assignment, but a higher frequency of misassigned reads (see Table S2). Consequently, it may potentially produce a higher number of false positives for minoritarian DTUs in the sample than for the other reference sets. The 97.5 reference set failed for five strains to assign DTUs, although the number of misassigned reads was considerably lower. Instead, the 95% reference set failed to typify only one strain, but it had a considerably lower rate of false positives than the 85% reference set. Consequently, we selected this threshold for most analyses. The following section has been rewritten to show the criteria selection for different reference sets and clarify the goals of the reference sets:

*Six sets of reference sequences were constructed based on the similarity thresholds (85%, 87.5%, 90%, 92.5%, 95%, and 97.5%). To evaluate the suitability of the reference sets for typing, each strain was retyped using a reference set (n – 1) constructed by excluding the sequences of the strain to be typed. Proportions of reads unassigned to any DTU, reads correctly assigned to the DTU of the strain (true positives), and reads erroneously typified to another DTU (false positives) were calculated (Table S2). In addition, the frequencies of strains that were correctly and incorrectly assigned were calculated (Table S2). As expected, higher thresholds imply higher specificity in read assignation, although it also implies less sensitivity for DTU assignation to strains (see the 97.5% reference set that failed to assign DTU to five strains in Table S2). We selected the 95% reference set because it allowed a lower false-positive rate for DTU assignment of reads, in spite of failing to genotype only one TcIII strain. Instead, all n – 1 reference sets constructed with an 85% similarity threshold were able to correctly typify the strains with their corresponding DTU, that is, most of the reads clustered with references of the same DTU. However, the higher rate of false-positive reads in this reference set (Table S2) may discourage its use in the detection of secondary DTUs in a sample. The proportion of reads clustered for each DTU using 85% and 95% reference sets is shown in Figure 2.*

Maybe I'm missing something here? I was perhaps expecting a number of figures (supplementary of course) similar to Fig3 but where 1) query genome is one strain/isolate per figure; and 2) the Figure shows both panels A + B (percentage reads, percentage of bases) for _all_ strains and isolates that make up the 95% reference set. In a hypothetical case of e.g. using CL-Brener (IPB/CSIC, IonTorrent, see below) as query, this figure would show the percentage of reads and bases matching each of the strains in the reference dataset. From here one should hopefully be able to see that CL-Brener matches itself and also maybe the other CL-Brener instance (UFMG/Brazil, Illumina) first (top-ranked) and then down maybe another TcVI strain, etc. This type of figure should show sub-DTU level matching of individual strains (if I understood clearly the aim of the 95% reference set).

Thank you for your insightful comment. In this manuscript, our primary aim was not to delve into the intricacies of intra-DTU diversity or clustering but rather to introduce a DTU typing method that could be efficient for biological samples. However, we recognize the importance of the observations you made and have now incorporated Supplementary File 2 to highlight sub-DTU level matches in the genome data, as you suggested. It can be observed that related strains within a DTU share a similar pattern in the heatmap. This observation echoes the data presented in Figure 1. Given these findings, our method holds the potential for elucidating intra-DTU relationships among strains. However, for more comprehensive clustering at the intra-DTU level, we acknowledge the necessity of increasing the number of strains to achieve conclusive subdivisions. We anticipate addressing this more extensively in the future, as we will expand our database with more strains.

It is important to highlight that some genomes matched better with other strains than with themselves (new heatmap provided in Supplementary File 2). This is because mHVR sampling was not homogeneous among the strains and some strains had a higher sequencing depth than others. For example, the CL-Brener genomes shared more mHVR clusters with LL015P68R0cl4 than with the CL-Brener strain in the mHVR dataset. This is because LL015P68R0cl4 is close to CL-Brener, but the number of reads of the former was approximately 71 times higher than the latter (See Supplementary Table 1).

Considering the above, we have included the following sentences in the results:

In addition, the proportion of mHVR clusters shared between different genomes and strains in the 95% reference dataset was addressed (Supplementary File 2). Different patterns were observed within some DTUs for different genomes, suggesting potential utility for intra-DTU typing.

And in the discussion:

In addition, the 95% reference set also showed different mHVR composition patterns within some DTUs, which highlight their potential use at intra-DTU typing. Future studies encompassing a broader range of strains from diverse geographic regions and dedicated reference sets for each DTU will be required.

4. Genome data used

Table 2, page 12, lines 146-148.

Why were these genomes selected? Was there any rationale to omit other genomes? This seems strange because, there are a number of Sylvio X10 genomes (DTU TcI) sequenced by different groups. Similarly for Dm28c (DTU TcI), and TCC (a CL-derivative strain), amongst others. Having the same strain/isolate sequenced independently would provide robust validation of the methodology and the developed reference datasets. Please clarify.

We appreciate your comment. Genome selection criteria were established as follows. First, we selected strains with raw reads deposited in publicly accessible databases. Consequently, we did not include the Sylvio X10 strain because raw reads for this genome were not available in public databases (only processed contigs are available). Furthermore, it was a prerequisite for the datasets that no fragment size selection was made before sequencing because minicircles have short size (1.4 kb) and they are discarded. Consequently, certain strains, TCC (PacBio sequencing) and Dm28c (Nanopore sequencing) were not included in our analysis because DNA fragments shorter than 10 kb were removed before sequencing in such projects. In addition, non-typified strains, experimental hybrids, various TcI genomes, and clones were present; however, our purpose was to analyze a representative set of DTUs diversity. Furthermore, we have now included in our analyses a Dm28c sequencing project with Illumina raw reads. Such data we erroneously omitted in the previous version. By using genomes from strains that were also part of the 95% reference set allowed us to affirm the efficacy of the implemented methodology and the integrity of the developed reference datasets.

To clarify this matter, the following paragraph has been added to the Materials and Methods section:

*“To evaluate the 95% reference set, raw sequences from different genome-sequencing projects were downloaded from the NCBI SRA database to evaluate a representative genome set of DTUs diversity. Considering the short size of minicircles, only genome projects with no fragment size selection previous sequencing were analyzed. Furthermore, the genomes analyzed were reported as previously typified.”*

Also here, what is the difference between the two CL-Brener genomes and why did you include these two? I see one is from the IPB/CSIC (Spain), sequenced using IonTorrent. And the other is from UFMG (Brazil), sequenced using Illumina. Besides clarifying why the authors included two datasets from the same strain, maybe the authors would like to expand on analyzing the ability to typify strains using either IonTorrent vs Illumina data?

In response to your query concerning the CL Brener strain, it is noteworthy that we considered the availability of genomic data generated through distinct technologies, such as Illumina and IonTorrent, retrieved from various databases. This allowed us to corroborate our typing workflow using genomic information derived from the two distinct sequencing platforms.

To provide further clarity on this aspect, we have incorporated the following information below the Table 2 in the Materials and Methods section.

*“^1^Sequenced with an Illumina HiSeq 2000.*

*^2^ Sequenced with Ion Torrent.”*

Results

“Furthermore, both CL Brener genomes exhibited a high degree of concordance in their typing, despite having been sequenced using different sequencing technologies.”

Reviewer #3: The rationale of the study is appropriate, the high sequence diversity and copy number of the high variabLe region of the minicircles of T.cruzi ( mHVR ) makes it a good marker for parasite genotyping at the DTU and infra_DTU levels. These aims are partially addressed by the experimental work because analyses has been done with DNA from reference strains, with datasets from T.cruzi genome projects and with mock samples simulating mixed DTUs. The authors have found adequate typing results with some limitations, in particular for the low number of strains for a given DTU such as Tc III. So, the sample size should be increased in particular for this DTU.

Statistical analyses are clearly described .

Which was the criterion to select 85% and 95% values as minimum similarity percentages instead of looking for the optimal minimum percentage to have two categories of classification : one that englobes all strains from a same DTU in a single cluster and accurately separate DTUs, and another one that could be used to distinguish subDTUs in a given population ?

Thank you for highlighting the potential of our method for direct typing, especially in the context of samples from chronic patients that commonly have a low parasite burden.

As previously mentioned in our responses, our selection of the threshold was based on our earlier publication (21). We conducted extensive evaluations to ascertain the optimal threshold for the mHVR clustering. The updated manuscript now provides a comprehensive comparison across different thresholds, ranging from 85% to 97.5% in increments of 2.5%. Our evaluations revealed that while the 85% threshold demonstrated better sensitivity for DTU assignment, it also lead to a higher frequency of misassigned reads, which could potentially introduce a higher count of false positives for minority DTUs in the sample. In contrast, while the 97.5% set was more stringent, leading to reduced misassignments, it did not assign DTUs for several strains. The 95% reference set emerged as a balanced choice, offering a significantly reduced rate of false positives when compared to the 85% set, and only failing to typify one strain. The detailed criteria for our selection and objectives of these reference sets have been rewritten in the revised manuscript.

In the broader context of our work, our primary goal remains was to introduce an efficient DTU typing method tailored to biological samples. We agree with your observation and have made provisions in our manuscript, such as the inclusion of Supplementary File 2, to show sub-DTU level matches. Indeed, the patterns seen in the heatmap and Figure 1 support the potential of our methodology to shed light on intra-DTU relationships among strains. We aim to further refine our approach by expanding the database to include more strains, thereby facilitating a more comprehensive insight into intra-DTU dynamics.

We hope that these clarifications address your concerns, and we appreciate the thorough feedback that significantly contributed to the improvement of our work.

In my opinion, the work would significantly enrich if this typing algorithm is applied to a panel of true biological samples already characterised using a previously reported method, to be able to detect degree of agreement and discordances.

Thank you for your constructive feedback on enhancing our study by validating our typing algorithm using previously characterized biological samples. We understand and recognize the value of such an assessment, and accordingly, we have expanded our manuscript to address this crucial aspect.

We have added a new section titled "Usefulness on blood samples of infected patients". In this section, we deep sequenced the mHVR amplicons obtained from 28 blood samples from infected patients enrolled in a previous study (see reference 34). These samples were evaluated using our typing algorithm, and the results were compared with those obtained previously by Southern blotting using mHVR probes.

The key findings of this evaluation are as follows:

Our amplicon sequencing method identified infecting DTUs in all samples (100%, 28/28), whereas the Southern blot method detected DTUs in 79% (22/28) of the samples. Both methodologies predominantly identified TcV as the most common DTU. We observed an overall concordance rate of 82% between the two methods with a kappa index of 0.24, indicating a fair level of agreement. There were some discrepancies in the detection of TcI and TcVI between the two methods. Notably, our method exhibited a higher sensitivity for TcI detection. Some disagreements regarding TcVI assignment were observed between both methods, which are discussed in the results section. The 85% reference set showed full concordance with the 95% set for the detection of primary DTU in the samples, but it indicated a higher prevalence of secondary DTUs, as we anticipated.

These results highlight the utility of mHVR amplicon sequencing for the characterization of blood samples, offering insights into its strengths and areas of divergence from traditional methods. By incorporating these comparisons, we believe that our study provides a more comprehensive and validated view of the applicability of the proposed typing algorithm to real-world scenarios.

The main changes to the manuscript regarding typing on blood samples are detailed below:

Materials and methods

*Strains and blood samples*

*DNA from 52 T. cruzi strains belonging to the six main DTUs was analyzed in this study (Table 1). Sequences of ten additional strains previously analyzed by Rusman et al. (21) were also included. Twenty-eight blood samples were obtained from a previous cross-sectional study conducted in February 2010 in El Palmar (27° 40 32,700S; 61° 340 19,900W), a settlement located in the 12 de Octubre Department, Chaco Province (Argentina)(34). The protocol was approved by the Bioethics Committee of the Faculty of Health Sciences at the National University of Salta, Argentina. Blood was preserved in guanidine-EDTA buffer (Five milliliters of blood mixed with an equal volume of a solution of 6 M-HCl and 0.2 M EDTA). A standard phenol-chloroform method was used for DNA extraction.*

*mHVR sequencing*

*To generate mHVR libraries from the blood samples, two consecutive PCR reactions were performed, each with a volume of 15µl. The first reaction mixture included 200nM of modified primers 121 and 122 described by Rusman et al. (21), 3µl of DNA, 0.375U of Fast Start High Fidelity Enzyme Blend (Roche), 1X buffer supplied with the enzyme blend, 4.5mM of MgCl2 (Roche), 5% DMSO (Roche), and 0.2mM of PCR grade nucleotide mix (Roche). This PCR protocol started with an initial denaturation for 3 min at 94°C, followed by two cycles of 97.5°C for 1 min and 64°C for 2 min. Then, 33 cycles of 94°C for 1 min and 64°C for 1 min were run, with a final extension at 72°C for 10 min. For the second reaction, which aimed to incorporate barcodes into the first reaction amplicons, the mixture contained 200nM of each barcode, 2µl of the primary amplicon, 0.375U of Fast Start High Fidelity Enzyme Blend (Roche), 1X buffer supplied with the enzyme blend, 4.5mM of MgCl2 (Roche), 5% DMSO (Roche), and 0.2mM of PCR grade nucleotide mix (Roche). The protocol for this reaction was as follows: initial denaturation for 3 min at 95°C, followed by eight cycles of 95°C for 30 s, 55°C for 30 s, and 72°C for 30 s, ending with a final extension at 72°C for 5 min.*

Results

*Usefulness on blood samples of infected patients*

*Building upon a prior study that examined the prevalence of different DTUs in blood samples from infected patients (34), we conducted deep amplicon sequencing of the mHVRs in such samples. The number of reads acquired for each of the twenty-eight samples, along with their corresponding DTUs, determined by using the 95% reference set, can be found in Supplementary File 3. These findings were compared with the Southern blot analysis using mHVR probes performed previously in such samples. Remarkably, amplicon sequencing identified at least one infecting DTU in all the samples (100%, 28/28), even in those with a low number of reads (Figure 6A and Supplementary File 3). Instead, the Southern blot method detected a DTU in 79% (22/28) of the samples (Figure 6A). Both techniques predominantly identified TcV as the most prevalent DTU, with frequencies of 27/28 and 22/28 for amplicon sequencing and Southern blotting, respectively (Figure 6B). A concordance rate of 82% was observed, and a kappa index of 0.24 indicated a fair level of agreement between the methods. In addition, neither method detected the presence of TcII or TcIII in any sample. These results clearly showed that mHVR amplicon sequencing can be implemented in blood samples. Although TcI and TcVI were less prevalent, there was a noticeable discrepancy in their prevalence between the two techniques. Amplicon sequencing revealed a high prevalence of TcI compared to TcVI. For TcI detection, the concordance was 64% (kappa = 0.05), with most of the identifications attributed to amplicon sequencing (10 versus 2). Conversely, TcVI was detected more frequently using the Southern blot method than amplicon sequencing, with counts of 14 and 8, respectively. There was a notable discordance in the detection of TcVI between the two techniques (kappa = -0.15). As predicted, the 85% reference set was fully concordant with the detection of the main DTU in the sample when compared to the 95% reference set. However, a higher rate of secondary DTUs was also observed (Supplementary File 3).*

*Discussion*

*We further assessed the amplicon sequencing efficacy using blood samples and found it to be proficient in assigning DTUs, even with a limited number of reads. When compared against Southern blotting using mHVR probes, our method showed overall good concordance. However, certain discrepancies were noted, particularly regarding the secondary DTUs present in the samples. Amplicon sequencing identified a higher prevalence of TcI than Southern blotting. This was expected because the TcI mHVR probe for Southern blotting was built with mHVR amplicons of a unique TcI strain (34). Instead, amplicon sequencing was based on sequences from 26 TcI strains, which enhanced its sensitivity. Conversely, TcVI was detected more frequently by Southern blotting than by our amplicon sequencing method. This result was unexpected because TcVI has a relatively low genetic diversity, and consequently, it would be expected to be fairly represented in the 95% reference set. It is important to note that the specificity of the Southern blot method was evaluated with a reduced dataset (less than 20 strains) (34) and that hybridization can be sensitive to probe incubation conditions. Consequently, the discordance may be attributed to the cross-reaction of the mHVR TcVI probe in the Southern blot.*

--------------------

<b>Results</b></br></br>

-Does the analysis presented match the analysis plan?</br>

-Are the results clearly and completely presented?</br>

-Are the figures (Tables, Images) of sufficient quality for clarity?</br></br>

Reviewer #1: The results are clear and organized according to the aim of the study. Images and tables are correctly visualized and clear.

Reviewer #2: -Does the analysis presented match the analysis plan?

YES

-Are the results clearly and completely presented?

YES

-Are the figures (Tables, Images) of sufficient quality for clarity?

Figure 2. Font / letter size of axes labels (e.g. "Strains"; "Set 85%", "TcI", "TcII") is disproportionately big in comparison with the tiny size of text (unreadable at default zoom level) containing strain / isolate names (e.g. "862021", "Armadillo1975", "Colombiana")

We have modified the labels of figure 2 as required.

Figures 3 & Supplementary Figure 1. Are these two the same? What is the difference between these two?

Please note that Figure 3 and Supplementary Figure 1 display different results. Figure 3 presents the results of the mapping analysis, considering a coverage of 270 bases and a minimum depth of 10X. On the other hand, Supplementary Figure 1 illustrates the same analysis, but with a coverage of 170 bases and a minimum depth of 10X. This has been explained in the figure legends. Increasing the minimum coverage reduced the number of sequences in the reference set that were mapped. Consequently, it is useful to address DTU assignments to different coverage when genome reads are used.

Reviewer #3: By comparing the diversity of mHVRs in 62 reference strains of the six main DTUs, at 80,000 reads of depth, the authors confirmed observations of previous works that employed other markers, as it is mentioned by them in the discussion section.

The 95% mHVR reference sequence set was evaluated for its suitability for typing sequences from genomic projects with good results.

Authors demonstrated that the representative sequence sets at 85% and 95% accuracy were both able to identify mock samples. It would be nice to add at least a panel of biological samples to be able to characterize better the limitations of the technique in the presence of host DNA of different procedences and in real mixed infections, for example with triatomines coinfected with different DTUs. For now, the usefulness of the 95% reference set in such an application is an hypothesis.

Thanks. We recognize the importance of validating our methodology in the context of true biological samples, especially in cases involving mixed infections. As stated above, we expanded our analysis to evaluate the performance of our method by using blood samples from infected patients. Our findings showed that mHVR amplicon sequencing successfully identified DTUs in all samples. Although there was notable agreement with the Southern blot results for the prevalent DTU, we observed certain discrepancies for the less common DTUs, providing valuable insights into potential areas for refinement.

Furthermore, this validation underscores that our method can effectively work in the presence of host DNA, as observed in actual patient samples. While we agree that evaluating triatomines coinfected with different DTUs would further enhance our understanding, our current results serve as an essential step, providing a robust foundation for establishing the potential of our method in practical applications.

As DTU 1 is so variable and distributed into different clusters, could be proposed from these data, together with data from others ( for example studies using microsatellites) , that DTU I actually might be conformed by more than one DTU ( eg. Ia, Ib...I n?) while DTU V and VI, for example are much more homogeneous ?

Our research clearly shows that some DTUs, such as TcI, have a much greater genetic variety than others, such as TcV and TcVI. This is easily seen in Figure 1 and the new Supplementary File 2. Identifying diversity is one thing but breaking it down to smaller and supported groups within DTUs is a significant challenge. To achieve this, it is essential to have access to a larger number of reference strains of these DTUs from different geographic regions and countries. In addition, such strains should be analyzed by different markers. In this manuscript, few strains were analyzed by SL-IR or MLMT by others. Consequently, it was not possible to conclude about sub-DTU clustering. However, we are optimistic that our approach looks promising, especially as we (and others) add more reference samples over time to the reference sets.

Given the importance of this topic, we have added a point in our discussion: *“In addition, the 95% reference set also showed different patterns of mHVR composition within some DTUs which highlight a potential use at intra-DTU typing although future studies encompassing a broader range of strains from diverse geographic regions and dedicated reference sets for each DTU will be required.”*

Figures

Labels of strains in Figures 2 and 3 need better resolution to be clearly read

We have modified the labels of Figure 2 and 3.

--------------------

<b>Conclusions</b></br></br>

-Are the conclusions supported by the data presented?</br>

-Are the limitations of analysis clearly described?</br>

-Do the authors discuss how these data can be helpful to advance our understanding of the topic under study?</br>

-Is public health relevance addressed?</br></br>

Reviewer #1: The conclusions are in accordance with the results and limitations are clearly expressed by the authors. These limitations are presented as future objectives of the group.

The scope of the study was not only the generation of new data about T. cruzi but also build a database to enable the study of this strategy worldwide.

Reviewer #2: 5. Discussion

page 23, lines 357-360

"Moreover, the workflow was evaluated for its ability to typing samples derived from cultures, a set of complete genome data, under different simulated PCR conditions, and in the presence of more than one infecting lineage."

I don't like this sentence. I understand what the authors are trying to state, but I would split this sentence in two: one sentence for the cases where you've used experimentally determined sequences (sequencing samples from cultured strains; sequences obtained from the public domain); and one for the artificially simulated samples (simulated PCR conditions; simulated co-infections). This way it should be more clear to readers.

We modified this as follow:

“*Firstly, we evaluated the workflow for its ability to genotype samples obtained from cultures and sequences sourced from the public domain such as genome data. Secondly, we assessed its performance under artificially simulated conditions, such as different PCR scenarios and in the presence of multiple infecting lineages. Finally, we addressed the workflow performance on blood samples derived from infected individuals*.”

Also I don't quite like how the PCR-results are discussed. Not trying to come hard on authors, you do say "simulated samples" often. So it should be clear. But I'd prefer to read "simulated PCR reactions" or "simulated PCR experiments" as this come closer to what the authors did, which is to 1) generate artificial DNA samples (templates) for PCR; and 2) run a simulated PCR amplification in the computer.

We clarified the paragraph as follow:

“*Our results further demonstrate that the 85% and 95% reference sets successfully and accurately typified the strains after a simulated PCR reaction which generates stochasticity on frequency of sequenced mHVRs. This was observed even under suboptimal simulated PCR conditions as low efficiency and few template DNA molecules*.”

I would also like to see a statement saying that this of course should be validated experimentally (detecting co-infections), maybe even using artificial (spiked) samples created in the lab (not in the computer) by mixing DNA from different strains. I am not asking that the authors do the real PCR validation for this Ms (great if you do, but not required for acceptance), only that you mention this fact.

We have added a paragraph to the discussion as suggested:

*“Our simulation-based approach provides valuable insights and demonstrates the potential for coinfection detection. However, further experimental validation is required to ensure reliability and applicability for detecting coinfections. Laboratory experiments using artificial samples created by mixing DNA from different known strains would be necessary.”*

6. Further Discussion

There are two additional issues I don't see discussed here.

mHVR amplicons are derived from mitochondrial DNA (kDNA). And inheritance of mitochondrial DNA may not follow nuclear DNA. Because other schemes and methods for typification of DTU are based on nuclear-DNA markers, I think it merits some discussion here on whether this is something that should be paid attention to, or not (and why?) I see the authors have some published analysis on this (ref #21). A brief discussion here would help readers put this in context.

We have added a paragraph to the Discussion as suggested. “*A potential drawback of our method is related to minicircle inheritance. Maxicircles from T. cruzi kDNA have been suggested to be inherited uniparentally, whereas we previously proposed that minicircles are inherited biparentally in hybrids (21, 49). If minicircles are inherited biparentally, they are expected to behave similarly to the nuclear genes. Therefore, we anticipate that the typing results will be similar to those obtained using other methods that use nuclear markers. However, when maxicircle markers are used for typing, divergent outcomes are anticipated in cases of hybridization or mitochondrial introgression. In this sense, it is crucial not to overlook the numerous instances of mitochondrial introgression that have been reported (50).*”

Amplicon sequencing is OK for cultured strains. And simulated PCR experiments are OK in this Ms to show something more towards application in a real world setting (clinical samples from patients). But this is not discussed much, and I think this is one of the major impacts perhaps in the long term. Maybe the authors would like to discuss something about performing PCR amplification of mHVR regions from clinical samples? Performance? Also maybe discussing this in the context of typical focal infections produced by T. cruzi where different infection foci in the body may activate at different times (e.g. see PMID:32799361). Is there something the authors could say on the applicability of this method to clinical samples? Future prospects?

As we mentioned above, we have now included genotyping of 28 blood samples from infected patients and we discussed the results in this new version of the manuscript.

Reviewer #3: Conclusions are supported as a feasibility study of the use of this typing algorithm but further studies using panels of biological samples and blind evaluation in the field will be necessary to demonstrate the potential of this proposal for epidemiological studies. A more robust work will be presented when the researchers will expand the reference sets of mHVRs to some DTUs and report the online database for automated data analysis.

Thank you for your valuable comment. We believe that the manuscript has been significantly enhanced by incorporating biological samples and comparing our approach with another typing method. Although we were unable to include additional TcIII strains in this study, the methodology we developed holds promise for DTU assignment. It serves as a foundational tool that can be further refined by the inclusion of more reference sequences in the future.

--------------------

<b>Editorial and Data Presentation Modifications?</b></br><br/>

Use this section for editorial suggestions as well as relatively minor modifications of existing data that would enhance clarity. If the only modifications needed are minor and/or editorial, you may wish to recommend “Minor Revision” or “Accept”.

Reviewer #1: (No Response)

Reviewer #2: MINOR ISSUES / COMMENTS

Introduction, page 7, lines 69-70

"Due to the low level of parasites circulating in the peripheral blood or infected tissues in chronically infected patients, most typing methods have limited sensitivity (24)."

ref #24 does not describe typing methods or performance at the task of discriminating DTUs, it only describes performance of PCR for _detection_ of T. cruzi using 6 DNA targets: kDNA, Sat-DNA, 24S, CO-II, SL-DNA, 18S.

The reference was replaced with a new one (24) and 25.

Methods, page 9, lines 99-100

"A Fragment Analyzer (Advanced Analytical Technologies, USA) was used to validate the libraries."

I have been unable to find this company. Please clarify which fragment analyzer was used in the study (model? Part number? manufacturer?). Alternatively describe the quality metrics used to assess nucleic acid integrity.

A detailed description was provided as follow: “*A 5200 Fragment Analyzer System (Advanced Analytical Technologies Inc.- Agilent, USA) was used to validate the estimated size of the libraries, as the average size of the mHVR amplicons was ~480bp.*”

Methods, page 11, lines 111-112 and 115-116

"outputs (seqs_otus.txt and the otu table) were filtered using filter_otus_from_otu_table.py"; "The most abundant sequence in each mHVR cluster was selected as the representative sequence using the “pick_rep_set.py” script"

Are both scripts available as part of the QIIME software or just the first one? Please clarify.

This was clarified as follow:

*“The outputs (seqs_otus.txt and the otu table) were filtered using “filter_otus_from_otu_table.py” script from QIIME v1.9.1 to discard those mHVR clusters with low abundance and conserving those that were observed more than five times. The datasets were first evaluated based on their ability to cluster strains of the same DTU. The most abundant sequence in each mHVR cluster was selected as the representative sequence using the “pick_rep_set.py” script from QIIME v1.9.1, with the other parameters by default.”*

Also here, lines 114-118, the text is confusing:

"The most abundant sequence in each mHVR cluster was selected as the representative sequence" (this implies there is only one representative sequence per cluster), but then the following sentence: "The output contained a representative set of sequences for each mHVR cluster..." is saying that there is _a set of sequences_ (several) for each mHVR cluster. Please clarify.

We clarified this as follows:

“The output is a FASTA file containing one representative sequence for each mHVR cluster with their corresponding cluster identifier.”

Methods, page 13, lines 154-155

"The reads were mapped against the reference set of mHVR at 95% similarity using BWA-MEM (36) with default parameters."

Please report the version of bwa-mem. Otherwise the phrase "with default parameters" may make no sense. Alternatively, please report and define these default parameters.

We added the BWA-MEM version as follow:

*“The reads were mapped against the reference set of mHVR at 95% similarity using BWA-MEM v.0.7.17.2 (38) with default parameters.”*

Methods page 14, lines 176-177

"First, the algorithm random samples s molecules from a multinomial distribution"

Maybe rephrase to "First, the algorithm samples s random molecules from a multinomial distribution"?

Modified to:

*“First, the algorithm samples s random molecules from a multinomial distribution (f_0_) according to (1)”*

Results, page 17, lines 224-225

To address the suitability of the analysis of mHVR sequences in a sample infected with T. cruzi, it was first evaluated [which?] mHVR sequences [were?] shared between different cultured strains.

Modified to:

“*To address the suitability of deep amplicon sequencing to genotype T. cruzi DTU in a sample, mHVRs of 62 strains from different DTUs were amplified by PCR and deep-sequenced.*”

Results, page 19, line 274

"Notably, all evaluated strains were accurately typing [typified?] using this approach"

Modified to:

“*Notably, all evaluated strains were accurately typified using this approach…”*

Results, page 19, line 297

"a DTU would be infecting a sample" infecting? what is the idea behind a DTU "infecting" a sample? Please clarify or use another word to convey your idea.

Modified to:

“*In other words, parasites from a certain DTU were considered present in a sample if their DTU-tag was the most abundant among the reads obtained from that sample.”*

Discussion page 25, lines 377, 379

"The 85% mHVR reference sequence set was able to typing all strains"

"the 95% mHVR representative sequence set was unable to typing one TcIII strain"

typify all strains? typify one TcIII strain?

Replaced by “genotype”.

Reviewer #3: ACCEPT

--------------------

<b>Summary and General Comments</b></br></br>

Use this section to provide overall comments, discuss strengths/weaknesses of the study, novelty, significance, general execution and scholarship. You may also include additional comments for the author, including concerns about dual publication, research ethics, or publication ethics. If requesting major revision, please articulate the new experiments that are needed.

Reviewer #1: I only have one concern about the typing method proposed by this study, and this is the cost of NGS in endemic areas of Chagas disease. Perhaps the authors can propose/think of a method based on the obtained data that is applicable in resource-limited contexts, which happen to coincide with the endemic regions of the disease.

Thank you for pointing this out. This development has an acceptable cost compared to other techniques that require amplification of multiple molecular targets for typing purposes. However, the obtained results did not reach the point-of-care (POC) technique. In this regard, no shared mHVR clusters were identified among all strains for most DTUs, preventing the establishment of a PCR scheme. In addition, implementation of the algorithm in specialized healthcare centers is feasible because of the limited bioinformatics knowledge required to perform the analyses on the Google Collaboratory platform. Furthermore, library generation in the laboratory entails straightforward PCR reactions. A significant advantage of this technique is evident in future epidemiological studies, in which a large volume of samples can be effectively analyzed.

Reviewer #2: The work by Rusman F et al is a nice and timely description of both methods and a reference data set that would allow other labs to perform amplicon sequencing of T. cruzi DNA samples to perform DTU assignment and/or detect co-infections.

The work is original, well-written, and provides a well-described method and code to perform strain and DTU-typing on DNA samples. The authors have validated their method and their reference databases by typing available genomes in the public domain as well as by performing simulated PCR experiments in the computer with different levels of sub-sampling of the original (template) DNA.

That said, I do have some comments and suggestions (see below). My recommendation is acceptance after revision. Congratulations to the authors on a clean and nice to read Ms!

We appreciate your comments, and we acknowledge your recommendations.

Reviewer #3: This is a well written interesting approach to improve genotyping of T.cruzi based on deep sequencing of the highly variable region of the multicopy minicircle DNA of T.cruzi , showing promising resolution when working with DNA from reference strains, data from genome projects and mock samples. Further studies using panels of biological samples and blind evaluation of this strategy in the field is still needed to demonstrate its applicability in epidemiological studies. A more robust work will be presented when the researchers will expand the reference sets of mHVRs to some DTUs and report the online database for automated data analysis.

Thanks for your time and comments. As stated above we have improved our manuscript based on your recommendations.
