# Supplementary material for: Wide reference databases for typing Trypanosoma cruzi based on amplicon sequencing of the minicircle hypervariable region
Source: PLoS Negl Trop Dis. 2023 Nov 13;17(11):e0011764. doi: 10.1371/journal.pntd.0011764 (PMC10681310; doi:10.1371/journal.pntd.0011764)
Supplement: Attachment — Submitted filename: Response to reviewers.docx [file pntd.0011764.s008.docx]

Reviewer's Responses to Questions

**Key Review Criteria Required for Acceptance?**


As you describe the new analyses required for acceptance, please consider the following:


**Methods**


-Are the objectives of the study clearly articulated with a clear testable hypothesis stated?

-Is the study design appropriate to address the stated objectives?

-Is the population clearly described and appropriate for the hypothesis being tested?

-Is the sample size sufficient to ensure adequate power to address the hypothesis being tested?

-Were correct statistical analysis used to support conclusions?

-Are there concerns about ethical or regulatory requirements being met?


Reviewer #1: (No Response)

Reviewer #2: Accept the responses provided, and the revised manuscript with the changes.

--------------------

**Results**


-Does the analysis presented match the analysis plan?

-Are the results clearly and completely presented?

-Are the figures (Tables, Images) of sufficient quality for clarity?


Reviewer #1: (No Response)

Reviewer #2: Accept the responses provided, and the revised manuscript with the changes.

--------------------

**Conclusions**


-Are the conclusions supported by the data presented?

-Are the limitations of analysis clearly described?

-Do the authors discuss how these data can be helpful to advance our understanding of the topic under study?

-Is public health relevance addressed?


Reviewer #1: (No Response)

Reviewer #2: Accept the responses provided, and the revised manuscript with the changes.

--------------------

**Editorial and Data Presentation Modifications?**


Use this section for editorial suggestions as well as relatively minor modifications of existing data that would enhance clarity. If the only modifications needed are minor and/or editorial, you may wish to recommend “Minor Revision” or “Accept”.

Reviewer #1: (No Response)

Reviewer #2: Regarding the new Supp File 1 and the additions to Methods and Results, I thank the authors for including these additional data and experiments. However I had trouble reading and figuring this all out, so I have one final suggestion (which the authors may ignore), which is to try and clarify this a little bit more for the readership. Below are my notes:

We sincerely appreciate your comment, the feedback and suggestions are highly valuable to us. We understand the importance of clarity and accessibility, particularly in the new Supplementary File 1.

line 252, methods
"PCR model was compared to empirical data of mHVR cluster abundances for two independent PCR reactions of the same sample (Supplementary File 1)."
maybe substitute "empirical" with "experimental"?

We have substituted ‘empirical’ with ‘experimental’ as suggested.

line 566. Supporting information (legend to figures?) "Supplementary File 1. Evaluation of the PCR simulating algorithm by comparison against PCR repetition of the strain LL015P68R0cl4."
The text in the response to us reviewers is more clear in explaining this, maybe add some more detail, e.g. as in
"Supplementary File 1. Evaluation of the PCR simulating algorithm by comparison against a duplicate experimental PCR of mHVRs from the LL015P68R0cl4 strain (TcVI)." Also, the legend in Supp File 1 says the strain is LL015P68R0 (add cl4?)

We appreciate your suggestion for improving the clarity and detail of the supplementary information. We changed the legend in the text as follows:

‘Supplementary File 1. Evaluation of the PCR simulating algorithm by comparison against a duplicate experimental PCR of mHVRs from LL015P68R0cl4 strain (TcVI).’

The legend in Supplementary File 1 was changed to reflect the strain name more accurately as 'LL015P68R0cl4'.

Supplementary File 1. What is the x axis in the plots? PCR cycles? What is the scale? There are no labels or tick marks for this axis. This should be clarified and added.

Thank you for your observation regarding the presentation of the plots on Supplementary File. The x-axis represents the mHVR clusters that were ordered based on the average cluster size (green line) between the real PCRs with a logarithm scale. We apologize for the oversight in omitting labels reference.

lines 424-425, 430, 434. There is something wrong with the kappa index here. The kappa index is used many times but it is not defined anywhere. Is this Cohen's Kappa? If so, it is confusing that there is a concordance rate of 82% with a kappa index of 0.24 (which suggests low agreement between the two techniques). Is there something wrong here? or this at least merits some discussion. Maybe it is just that percent agreements are not robust in comparison with cohen's kappa?

We appreciate your comments and observations. As the Reviewer suggested, we have included a sentence in the Discussion section about the difference between both agreement measures.

‘In particular, TcV detection had a high percentage concordance (82%), although with a low kappa index (0.239). It is important to consider that Cohen’s kappa accounts for chance agreement, meaning it adjusts for the agreement that would be expected just by chance. However, this index was influenced by the prevalence of DTU infection. If the prevalence of a DTU is either very high (as observed for TcV) or very low, chance agreement is also high and kappa is reduced accordingly (47). This explains the high agreement percentage and low kappa index for TcV detection.’

--------------------

**Summary and General Comments**


Use this section to provide overall comments, discuss strengths/weaknesses of the study, novelty, significance, general execution and scholarship. You may also include additional comments for the author, including concerns about dual publication, research ethics, or publication ethics. If requesting major revision, please articulate the new experiments that are needed.

Reviewer #1: The authors have addressed all the questions and suggestions from the reviewers and have made the relevant modifications to the manuscript

Reviewer #2: Accept the responses provided, and the revised manuscript with the changes.

--------------------

PLOS authors have the option to publish the peer review history of their article ([what does this mean?](https://journals.plos.org/plosntds/s/editorial-and-peer-review-process#loc-peer-review-history)). If published, this will include your full peer review and any attached files.


If you choose “no”, your identity will remain anonymous but your review may still be made public.


**Do you want your identity to be public for this peer review?** For information about this choice, including consent withdrawal, please see our [Privacy Policy](https://www.plos.org/privacy-policy).

Reviewer #1: No

Reviewer #2: Yes: Fernán Agüero
